# COVID-19 infection inference with graph neural networks

**DOI:** 10.1038/s41598-023-38314-3

**Published:** 2023-07-15

**Authors:** Kyungwoo Song, Hojun Park, Junggu Lee, Arim Kim, Jaehun Jung

**Affiliations:** 1 Department of Applied Statistics, Yonsei University, Seoul, 03722 Republic of Korea; 2 Artificial Intelligence and Big-Data Convergence Center, Gil Medical Center, Gachon University College of Medicine and Science, Incheon, 21565 Republic of Korea; 3 Department of E-Learning, Korea National Open University, 03087 Seoul, Republic of Korea; 4 Incheon Communicable Diseases Center, Incheon, 21554 Republic of Korea; 5 Department of Preventive Medicine, Gachon University College of Medicine, 38-13, Dokjeom-Ro 3 Beon-Gil, Incheon, 21565 Republic of Korea

**Keywords:** Viral infection, Experimental models of disease, Public health

## Abstract

Infectious diseases spread rapidly, and epidemiological surveys are vital to detect high-risk transmitters and reduce transmission rates. To enhance efficiency and reduce the burden on epidemiologists, an automatic tool to assist with epidemiological surveys is necessary. This study aims to develop an automatic epidemiological survey to predict the influence of COVID-19-infected patients on future additional infections. To achieve this, the study utilized a dataset containing interaction information between confirmed cases, including contact order, contact times, and movement routes, as well as individual properties such as symptoms. Graph neural networks (GNNs) were used to incorporate interaction information and individual properties. Two variants of GNNs, graph convolutional and graph attention networks, were utilized, and the results showed that the graph-based models outperformed traditional machine learning models. For the area under the curve, the 2nd, 3rd, and 4th order spreading predictions showed higher performance by 0.200, 0.269, and 0.190, respectively. The results show that the contact information of an infected person is crucial data that can help predict whether that person will affect future infections. Our findings suggest that incorporating the relationships between an infected person and others can improve the effectiveness of an automatic epidemiological survey.

## Introduction

The COVID-19 pandemic has shocked the world with the speed at which it is spreading and its extreme infectivity. The COVID-19 pandemic, which broke out in early December 2019 is still far from over as the number of infections and deaths continue to rise. COVID-19 has affected almost every facet of human life, including social, physical, environmental, and economic aspects. Several types of infectious diseases have a high degree of transmissibility, and epidemiological surveillance plays a critical role in identifying individuals who may be at increased risk of transmitting these diseases and in reducing the rate of transmission. The development of automated tools to support the conduct of epidemiological surveys is of great importance, as it can reduce the workload of epidemiologists and improve the overall speed and efficiency of the investigative process. In other words, an effective strategy to control the spread of infection and reduce the rate of transmission would be extremely beneficial.

Since February 2020, professional investigators have been conducting epidemiological surveys in Korea on various items (e.g., demographic information, person in contact, time of contact, moving lines, and symptoms) relating to persons who are or have been infected with COVID-19. According to the results of the epidemiological surveys, the epidemiological investigators classify the type of care for infected persons, i.e., treatment at home or in a medical institution. Although the COVID-19 confirmed cases remain significant in South Korea, the government continues to implement relatively high protection policies, including isolation measures.

In addition, with the results of aggregated epidemiological surveys, the government will be able to take certain preventive measures against further infection and establish new policies. The epidemiological survey data provided an opportunity to create a relationship map between major infection clusters and Incheon City, Korea; the results of the surveys allowed us to create a relationship map at various stages between the infected and the contacted persons.

**Figure 1:**
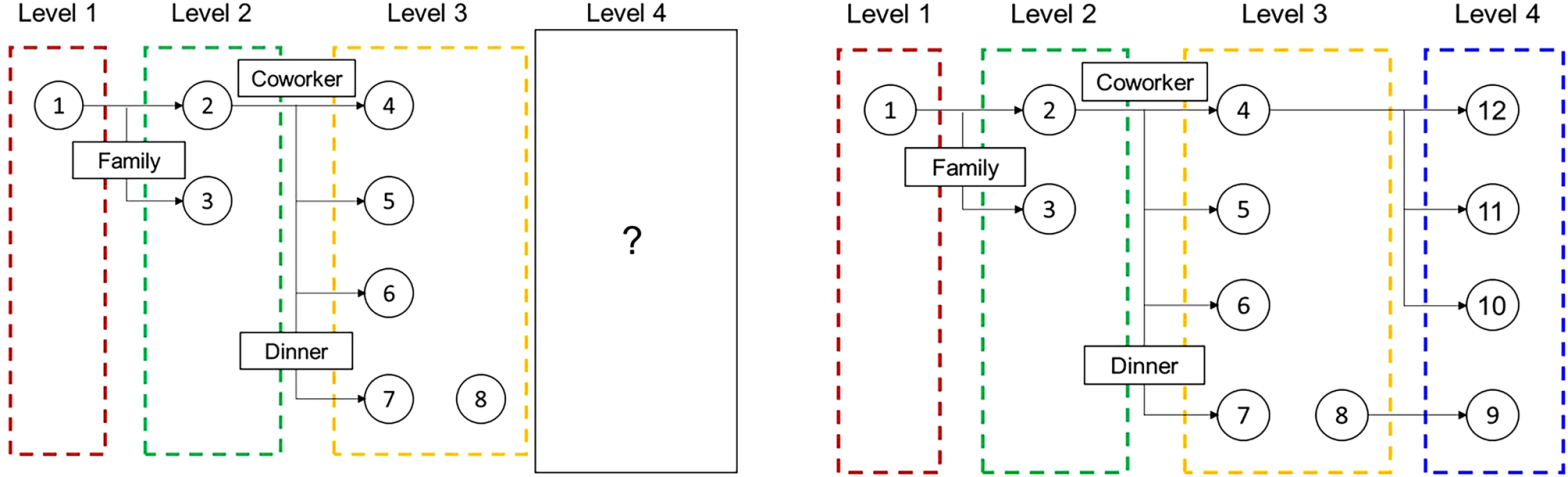
The visualization of infection inference for this paper. Each circle node and an edge represent a person and contact information, respectively. The rectangular box denotes the contact type for each person. The left and right figures denote the training set and test set, respectively. This study aims to infer whether the given person has an effect on the others when the others are unknown.

The objective of this study is to explore the possibility of the development of an automatic epidemiological survey. In other words, we propose a graph neural network-based algorithms to predict whether the specific confirmed cases influence to the additional infection in the future. Graph neural network is effective to capture the interaction information between confirmed cases and their own properties. Thus, we performed an infection inference for COVID-19 with graph neural networks, and Fig. 1 denotes the summarized results. We utilized the COVID-19 infection network data from Incheon City. As shown in Fig. 1, the dataset contains each person’s information and the relationship information between people. Our objective is to predict whether the infected person would affect the future infection of other people. For application in the real world, we assumed that the contact information between the current infected person and the future infected person was not accessible. In other words, we infer the effect when future contact information is not given for the realistic evaluation. Therefore, we conjecture that our method is applicable to real-time prediction.

## Methods

### Experimental settings

#### Dataset

**Figure 2:**
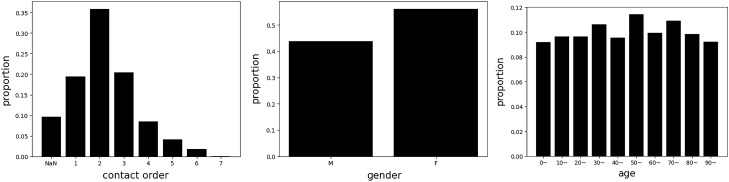
Histogram of the given dataset. Right denotes the relative frequency for infection order, and (NaN) denotes the missing feature. Middle and right denote the relative frequency for gender and age, respectively.

In this study, we utilized the dataset collected from Incheon City. The dataset contained data relating to the infection network for 1,678 people and 183 graphs. It included information in respect of the respondents’ relationships as well as the respondent’s characteristics. For each person, the dataset contained personal information, such as infection order, gender, age, and symptom-related information (Supplemental Table 1). Infection order refers to the degree of additional infections caused by one confirmed case. For example, if A infects B and B infects C, B and C are expressed as the 2nd and 3rd order infected person of A, respectively. Figure 2 describes the relative frequency of infection order, gender, and age. We constructed the dataset with actual contact information. In other words, we did not include individuals who had no contact history, even if two people were family members.

**Figure 3:**
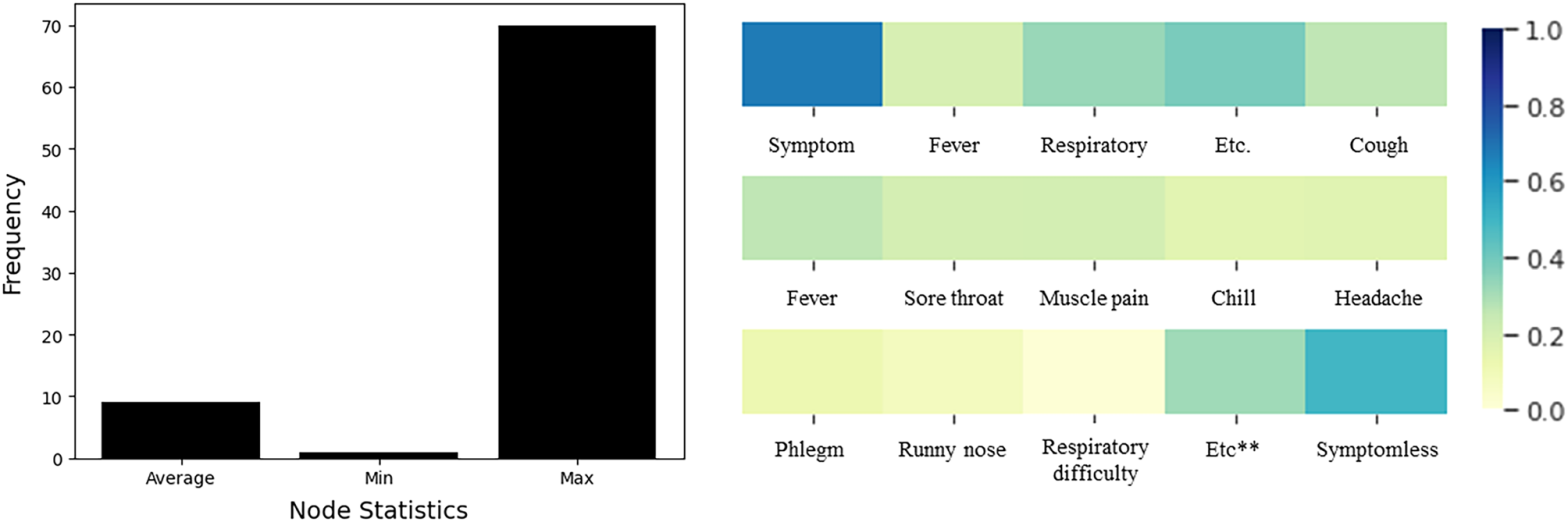
Histogram and heatmap of the given dataset. Left describes the average, minimum, and maximum node for the given graph. Right denotes the heatmap for each symptom. Given dataset has symptom records for each person, and we averaged them to create a visualization. 1.0 denotes the positive, and 0.0 denotes the negative.

**Figure 4:**
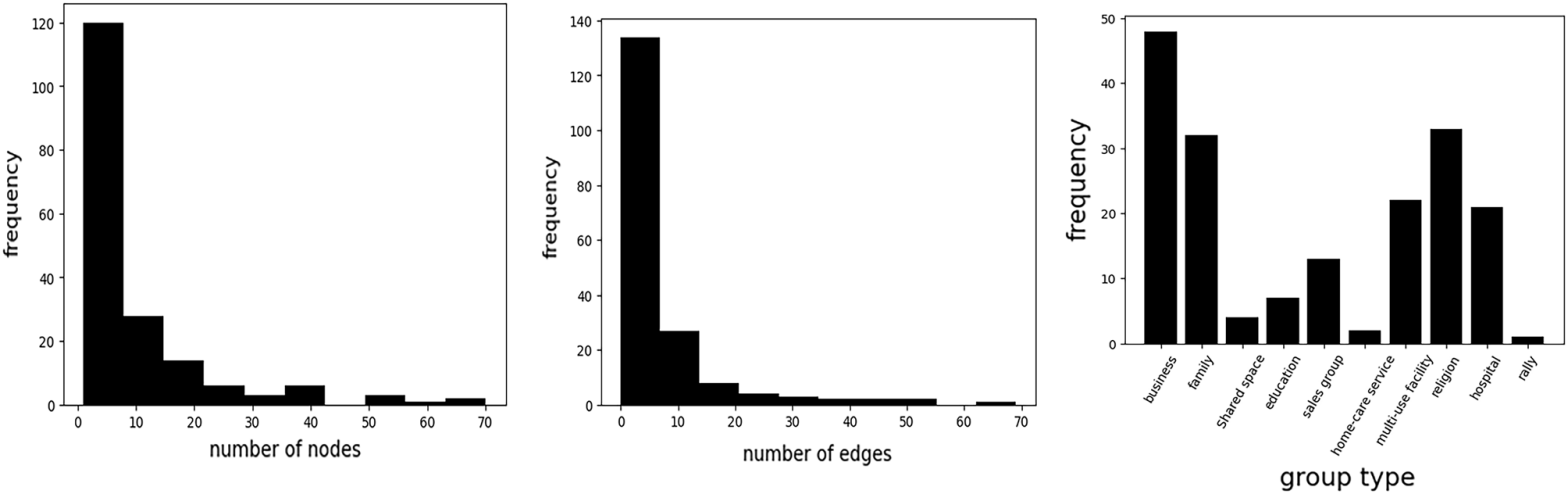
Histogram of the given dataset. Left and Middle describe the distribution of the number of nodes and edges in the dataset, respectively. Right figure denotes the frequency of group types. Our dataset contains diverse group-type dataset, such as business, family, and rally related infection networks.

Furthermore, Fig. 3 denotes the statistics for the graph size and the symptom-related information. The average graph size is 9.16, and each graph has different spreading patterns and routes, therefore, the size is diverse over the graphs. Figure 4 also denotes the dataset distributions, and our dataset contains diverse group-type dataset.

Our constructed COVID-19 data is a type of a scale-free network rather than a small-world network. Each node in the clusters or contact graphs have diverse degrees, and the degree follows the power law. Our constructed dataset follows P(k) ~ k^(− 1.798), where P(k) represents the fraction of nodes that have k edges.

#### Preprocessing

To analyze the dataset, we converted certain features into a binary value, and removed instances that had a missing value. For the gender characteristic, we represented male and female, as zero and one respectively. For the age characteristic, we converted the person’s age to zero if their birth year was less than 1982 and to one otherwise. In addition, we removed the small graphs that had less than three nodes.

As the objective of this study was to deduce whether the target person infects others or not and how many people we assumed that there was no additional future contact information for the target person. Therefore, we only utilized the 1st,…, n − 1th order’s people information to predict whether the target individuals had an effect on the nth order infection.

### Ethical approval

This study was approved by the Institutional Review Board of Gachon University College of Medicine, Incheon, South Korea (IRB No. GCIRB2021-434), and participant consent was waived by the ethics committee of Gachon University College of Medicine because the data involved routinely collected medical data that was processed anonymously at all stages. The study was conducted ethically according to the World Medical Association’s Declaration of Helsinki.

### Method Formulation

In this study, we utilized two GNN variants, graph convolution network (GCN), and graph attention network (GAT), to extract the strongest representation of the given dataset. Based on the representation, we predict the class of the given dataset. In this section, we introduce the general graph neural network structure, GCN, GAT, and classification in detail.

### Graph Neural Networks

GNNs are one of the most promising approaches to handle graph-structured data. GNNs provide the general framework to incorporate the properties of graphs by learning node embedding and edge embedding. The basic idea of GNNs is to handle the homophily assumption^1^. Each individual tends to bond with similar ones. GNNs reflect a homophily assumption with two phases, the message passing phase and readout phase^2^. The message passing phase indicates the information flow between the given node and its neighborhoods, and the readout phase is the information aggregation that comes from the message passing phase. It is noted that the message passing, and readout phase occurs for the given node and its first-order neighborhood. The restricted operation between direct neighborhoods represents the homophily assumption. To handle the multiple-order neighborhood, we stack GNNs deeply, thus encouraging the information flow between given nodes and their diverse neighborhoods.

**Figure 5:**
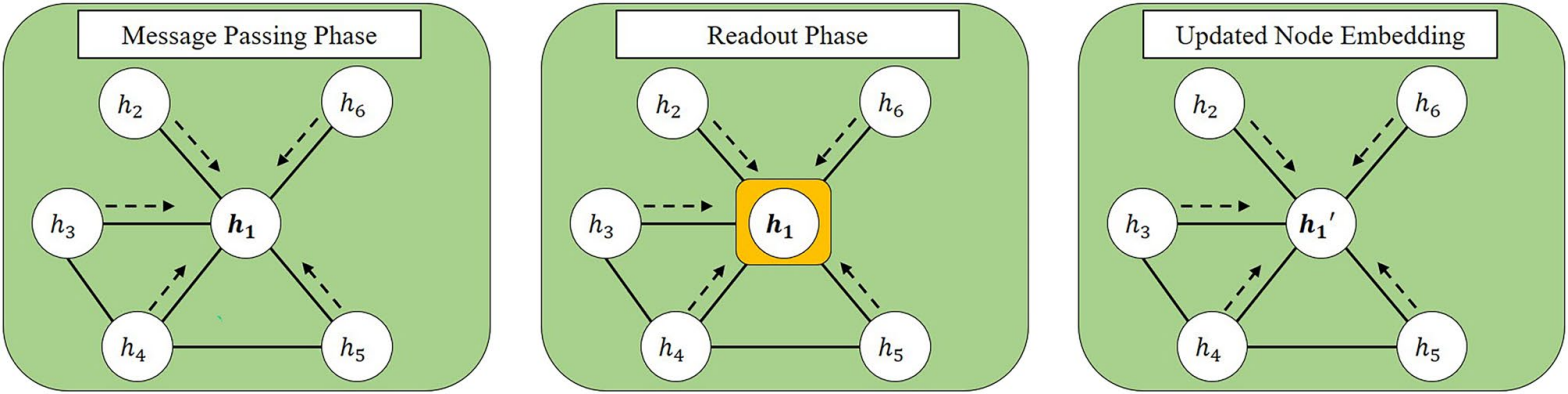
The illustration for GNN procedure for node embedding h_1. Left denotes the message passing phase, and it calculates the relationship between the given node embedding h_1 and its neighborhoods. The middle represents the readout phase, and it aggregates the relationship calculated in the message passing phase. After the message passing and readout phase, the node embedding for 1st node is updated from h_1 to h_1′.

Figure 5 denotes the illustration for the message passing phase and readout phase.

There are many variants of GNNs, and most variants adopt the message passing and readout phase framework. However, the specific algorithm of message passing, or readout phase induces the different GNNs. In this study, we adopt the GCN and GAT that utilize convolution and attention operation in the message passing phase, respectively.

### Graph Convolutional Networks

To handle the relationship between nodes, an effective message passing algorithm is necessary. GCN adopts the simple and effective algorithm, approximated spectral graph convolution to incorporate the neighborhoods’ information. The spectral convolution for graphs can be defined as a multiplication of filter ${\text{g}}_{{\text{w}}}$ and node embedding $h$ in the Fourier domain. We can represent the multiplication on the Fourier domain with the $U$, the eigenvectors of normalized graph Laplacian matrix.

$${\text{g}}_{{\text{w}}} *h = U{\text{g}}_{{\text{w}}} U^{T} h$$

However, the calculation of $U$ requires a large complexity burden. When the number of nodes is $N$, it requires $O\left( {N^{2} } \right)$ complexity. To alleviate the problems, GCN approximates the convolution operation with the first-order Chebyshev^3^ polynomials. The approximation induces the below-simplified form.

$${\text{g}}_{{\text{w}}} *h = W\left( {I_{N} + D^{{ - \frac{1}{2}}} AD^{{ - \frac{1}{2}}} } \right)h$$

It is noted that $D$ and $A$ denote a degree matrix and adjacency matrix, respectively.

### Graph Attention Networks

For the message passing phase, the traditional convolution operation in GCN is known to have difficulty in interpretability. Recently, attention mechanisms that focus on the important input features or hidden features have been widely adopted in the machine learning community. Graph Attention Networks (GAT)^4,5^ adopt the attention mechanism into the message passing phase. The attention mechanism is easy to interpret, and helps to focus on the important nodes.

GAT computes the attention logits between node $i$ and *j*—$e\left( {h_{i} ,h_{j} } \right)$—with each node embedding $h_{i}$ and $h_{j}$. GAT concatenates the node embedding $h_{i}$ and $h_{j}$, and it utilizes the linear projection with a learnable weight matrix $W$. In addition, GAT adopts the non-linear activation function $\sigma$ such as Leaky Rectified Linear Unit (LeakyReLU)^6^ to encourage the nonlinearity and representation power. The output value of non-linear activation is computed with vector ${\text{a}}$ to produce the scalar value, $e\left( {h_{i} ,h_{j} } \right)$.

$$e\left( {h_{i} ,h_{j} } \right) = {\text{a}}^{{\text{T}}} \sigma (W \cdot [h_{i} ||h_{j} ])$$

with logits $e\left( {h_{i} ,h_{j} } \right)$, GAT calculates the attention weights of node $j$ in terms of node $i$, α_ij_, with softmax function. The softmax function transforms logits to probability simplex, and attention weights can be interpreted as a probability. It is noted that the denominator of softmax function in GAT only handles the neighborhoods of node $i$. Similar to GCN, GAT with a single layer, also calculates the relationship between the node and its first-order neighborhood.

$${\upalpha }_{{{\text{ij}}}} = \frac{{\exp \left( {e\left( {h_{i} ,h_{j} } \right)} \right)}}{{\mathop \sum \nolimits_{{j^{\prime} \in N_{i} }} \exp \left( {e\left( {h_{i} ,h_{j} } \right)} \right)}}$$

Alternatively, GAT with the nth layer handles its nth order neighborhood. The first layer of GAT handles the relationship between 1st order neighborhood and updates its node embedding. The second layer of GAT calculates the attention between the node’s hidden features, the output of the first layer. Therefore, the second layer of GAT is able to handle the relationship between the node and its second neighborhood.

### Classification

For the infection inference task, learned node embedding and a classifier were necessary. GNN and their variants are effective for learning node representation. With learned node representation, we adopted a simple linear classifier to classify whether each node had an effect on the next round of infection or not. For the classification task, we adopted a cross-entropy loss that compared the ground-truth label $y_{i}$ and predicted label $\hat{y}_{i}$ for node $i$. In addition, we adopted $L_{2}$ regularization that minimized the magnitude of the parameters in GNN and the classifier, $\theta$, to encourage the reliability of our models^7^.

$$L\left( {y,\hat{y};\theta } \right) = - \mathop \sum \limits_{i = 1}^{n} y_{i} \log \hat{y}_{i} + \theta_{2}^{2}$$

## Results

### Baseline methods

In this study, we compared the graph-based model, GCN, GAT with the non-graph-based models. For baselines, we adopted logistic regression, support vector machine, and neural network.

Logistic Regression (LR)^8^: LR is one of the representative binary classifiers that outputs the probability of each class. LR formulates the log-odds with multiple independent variables.

Support Vector Machine (SVM)^9^: SVM induces the maximum-margin hyperplane. The hyperplane has the maximum distance for the nearest data instance for each class. SVM is known to be effective when one has a high-dimensional dataset.

Neural Networks (NN)^7^: A traditional machine learning algorithm is sensitive to feature engineering, and thus formulating a hand-crafted attribute is not insignificant. The neural network utilizes multiple non-linear transformations, and it induces a valid representation. For the classification task, we adopted NN + LR that extracted the significant characteristics with NN and classified them with LR.

### Experimental results

The objective of this study was to predict whether ${\text{n}} - 1{\text{th}}$ order’s infected person would affect the ${\text{nth}}$ order infection or not. To explore this diverse situation, we set ${\text{n}}$ as 2, 3, 4. Table 1 indicates the experimental results for the graph-based model and others. For evaluation metrics, we adopted accuracy (Acc.), the area under the curve (AUC), and the F_1_ score. GCN and GAT showed relatively improved performance compared to the LR, SVM, and NN. Notably, we did not utilize the future interaction information—the interaction between ${\text{n}} - 1{\text{th}}$ order infected person and nth order infected person. The results indicate that the previous contact information for a given infected person could be helpful to predict whether the person would have an effect on future infections. As shown in the Table 1, graph-based methods such as GCN and GAT show superior performance at higher-order prediction tasks. In terms of the 2^nd^ order case, there is only scarce graph-structured information; therefore, non-graph and graph-based algorithms do not show competitive results. Besides, the lower the order, the smaller the number of training data. The small number of datasets might incur all models having overfitting problems. When we compare the AUC metric between non graph-based model (LR, SVM, NN) and the graph-based model (GCN, GAT), the graph-based models improve the AUC by 0.200, 0.269, and 0.190 for the 2nd, 3rd, and 4th orders, respectively. These tasks have relatively dense interaction dataset, making graph-based methods effective in capturing the relational information between nodes.

**Table 1:** Graph neural network-based models show relatively high performance compared to the traditional machine learning methods.

Predicting orders	LR	SVM	NN	GCN	GAT
*2nd order prediction*					
Accuracy	0.6250	0.6250	0.6250	0.6250	0.6250
AUC	0.4000	0.5333	0.4000	0.5333	0.7333
$F_{1}$ score	0.7692	0.5714	0.7692	0.7692	0.7692
*3rd order prediction*					
Accuracy	0.9038	0.9231	0.9135	0.9327	0.9327
AUC	0.6276	0.4466	0.6224	0.8971	0.7982
$F_{1}$ score	0.9495	0.9600	0.9548	0.9645	0.9648
*4th order prediction*					
Accuracy	0.9178	0.7808	0.7534	0.8904	0.9315
AUC	0.4859	0.7465	0.7746	0.9648	0.6559
$F_{1}$ score	0.9571	0.8769	0.8548	0.9403	0.9645

*LR* logistic regression, *SVM* support vector machine, *NN* neural network, *GCN* graph convolution network, *GAT* graph attention network, *AUC* area under the curve.

In the 4th order prediction case, approximately 87.5% graphs are fully predicted accurately, while our model partially predicts the correct answer in the remaining 12.5% graphs. Our model performs well on the overall dataset, but it works even better for dense graph-structured datasets that have relatively rich interactions between nodes.

**Figure 6:**
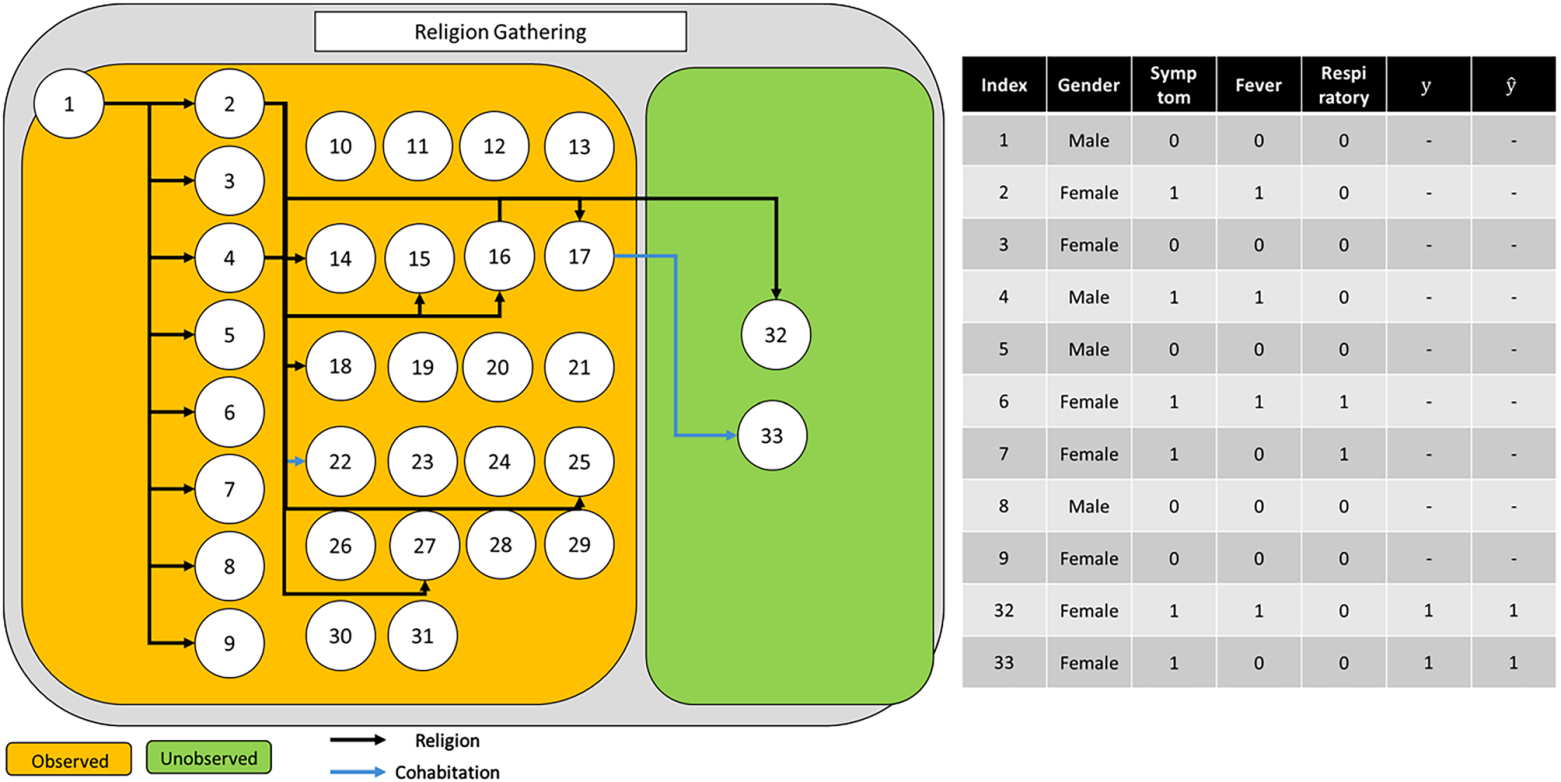
Example of an infection network about religion gathering. Given the observed nodes, our model, GAT-based infection prediction, predicts the infection accurately for all nodes.

To further investigate the effectiveness of our method, we provide additional qualitative analysis. First, we provide the correct and failure cases of our methods in Fig. 6, Supplementary Figure 1, and Supplementary Figure 2. Each figure contains the infection networks as well as the node (patient) property. In many cases, our method predicts the value of whether the specific node influences additional confirmed cases or not.

**Figure 7:**
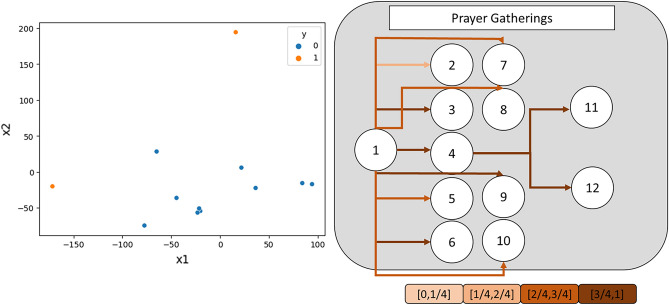
Example of an t-SNE visualization and attention weight visualization of “Prayer Gatherings” infection networks.

In addition, we provide t-SNE visualization and attention weight visualization to help interpret our methods. The t-SNE visualizations show the learned node embeddings from our methods, and they demonstrate that our method effectively separates nodes by their values, projecting them onto a linearly separable space. This separation is distinguished by a simple linear decision boundary. Meanwhile, the attention weight visualizations demonstrate that our method automatically learns the adaptable weights or importance of each node, which change dynamically depending on the node and graph properties. The attention weights vary across the node embeddings, and the color-coded visualizations help to illustrate these variations. Figures 7, Supplementary Figure 3 and Supplementary Figure 4 show examples of these visualizations.

## Discussion

The pandemic spread of COVID-19, is not over and the number of infections and deaths is still rising. COVID-19 is causing a lot of social and economic damage, and strategies are needed to control the spread of the infection and reduce the rate of its spread.

In Korea, from February 2020, professional investigators will conduct epidemiological surveys on various groups of people infected with COVID-19, and quarantine and manage infected people according to the results and symptoms of the surveys. Among the information collected through these epidemiological surveys, it was possible to create a relationship map between the infected and the contacts through information such as contacts, contact times, and movement routes.

The goal of this study is to predict whether a person infected with COVID-19 will affect the future infection of others by using the relationship map and for this, we used GCN and GAT that two variant models of GNN. To evaluate the two models we developed, a comparison was performed based on three indicators (accuracy, AUC, $F_{1}$ score) with traditional machine learning models (LR, SVM, NN), rather than graph-based models.

The graph-based models developed in this study showed better performance in predicting future infections compared to traditional machine learning models. These results, in addition to confirming the performance of the graph-based model, show that contact information for an infected person is data that can help predict whether that person will affect future infections.

Handling time-varying datasets is crucial for accurate predictions. To achieve this, we gather important time-invariant features, such as symptom records, to enhance our predictions of the number of additional infections. Additionally, graph neural networks implicitly incorporate previous interactions and their changes, which enables our graph-based models, such as GCN and GAT, to effectively predict future outcomes with time-varying and stochastic features. Our graph-structured dataset incorporates the short-term dynamics; therefore, graph neural network-based methods might handle the short-term time-varying information or features better than traditional non graph-based methods.

Graph-based models are advantageous as they incorporate the interaction between nodes and rich relational information, which is helpful in predicting values. We can utilize even more relational information in the third and fourth order as compared to the second order. Thus, we posit that graph-based models have a significant advantage for higher-order prediction tasks.

Our study has some limitations. First, the findings are based on data from a limited number of datasets. Second, this model does not consider the mutation of the virus, although it seems that it is possible to update the model through correction and learning using the basic infection reproduction index for every future mutation. Third, it is important to handle the time-varying or stochastic features for the precise prediction. However, our graph-based models, such as GCN and GAT, have difficulty capturing the long-term time-varying information or features effectively. To incorporate the long-term time-varying information, it is necessary to utilize temporal graph neural networks or updatable graph neural networks. Therefore, we will explore the use of temporal graph neural networks and updatable AI for COVID-19 interaction research in the future.

In the field of COVID-19 research, GNN has been mainly used to develop diagnostic tools through image processing such as CT and chest X-ray^10,11^.

In 2021, there was a study in Korea that created an infection spread network using open data and analyzed its structural characteristics^12^. However, there was a lot of information that the actual contacted person had not confirmed. To solve this problem, when another person was identified in the infection route of one person, the method assumes a connection between them, and there was a limit to data accuracy.

Besides, there are additional COVID-19 detection studies^13–15^. They utilize artificial neural networks^13^, decision trees^14^, and diverse data mining algorithms, such as Ada-boost and sequential minimal optimization^15^. They show comparable performance, but we conjecture that graph neural network-based algorithms are necessary to incorporate the infection interaction network as well as patients’ own features well.

In this study, in collaboration with the government, an infection network based on the actual epidemiological investigation results was directly produced to increase the accuracy of the data, thereby increasing the reliability of the spread prediction.

Pandemic prevention requires accurate epidemiology to minimize the influence of each infected person. However, it is difficult to evaluate each person’s influence manually when the number of infections becomes substantial. This study sought to lessen this burden by analyzing the potential influence of infected persons automatically. The model we developed predicts the future infection influence of a given person based on their previous contact information. It will be able to assist in determining whether a person should be quarantined by predicting the probability of the virus spreading to the next person. The experimental results show that the graph-based machine learning model is relatively effective compared to the non-graph-based models. We suggest that additional extensive experiments on a large dataset are necessary to verify the superiority of the GNN. Further, we believe that large-scale experiments, model development, and better interpretability is needed.

## Conclusion

In this paper, we predict the infection influence given the infection graph networks. Different from the previous works, our method incorporates the interaction information between patients as well as patients’ own features, such as gender, age, and symptoms. Our methods show superior performance with rich interaction information compared to the baseline methods. As a future work, we will investigate the scalable updatable graph neural network algorithms to handle the time-varying important features effectively.

## Supplementary Information

Supplementary Information.

## Data Availability

The data that support the findings of this study are available from Incheon Communicable Diseases Center (http://www.icdc.incheon.kr/) but restrictions apply to the availability of these data, which were used under license for the current study, and so are not publicly available. Data are however available from the authors upon reasonable request and with permission of Incheon Communicable Diseases Center.
