# Supplementary material for: COVID-19 infection inference with graph neural networks
Source: Sci Rep. 2023 Jul 15;13:11469. doi: 10.1038/s41598-023-38314-3 (PMC10349841; doi:10.1038/s41598-023-38314-3)
Supplement: Supplementary file 1 — Supplementary Information. [file 41598_2023_38314_MOESM1_ESM.pdf]

Supplementary material

COVID-19 Infection Inference with Graph Neural Networks

Kyungwoo Song, PhD[†1]; Hojun Park, PhD[†2]; Junggu Lee, BE[3]; Arim Kim, PhD[4]; Jaehun Jung, MD, PhD[2,5,*].

[1] Department of Applied Statistics, Yonsei University, Seoul, 03722, Republic of Korea.
[2] Artificial Intelligence and Big-Data Convergence Center, Gil Medical Center, Gachon University College of Medicine and Science, Incheon, 21565, Republic of Korea.
[3] Department of e-Learning, Korea National Open University, 03087, Seoul, Republic of Korea.
[4] Incheon Communicable Diseases Center, Incheon, 21554, Republic of Korea.
[5] Department of Preventive Medicine, Gachon University College of Medicine, Incheon, 21565, Republic of Korea.
[†]These authors contributed equally to this work as first authors.

***Corresponding author:**
Jaehun Jung, MD, PhD.
Department of Preventive Medicine, Gachon University College of Medicine
38-13, Dokjeom-ro 3 beon-gil, Incheon, 21565, Republic of Korea.
Tel: +82-10-6359-3201; Fax: +82-32-458-2608; E-mail: eastside1st@gmail.com

Contents

Supplementary Table 1. Epidemiological survey items
Supplementary Figure 1. Example of an infection network about family gatherings. Given the observed nodes, our model, GAT-based infection prediction, predicts the infection accurately. The yellow square denotes the observed nodes, while the green denotes the unobserved variable.
Supplementary Figure 2. Example of an infection network about pub. Given the observed nodes, our model, GAT-based infection prediction, predicts the infection accurately for nodes 2 and 4. However, our model predicts that node 3 causes additional infections, while the node 3 patient does not influence another infection.
Supplementary Figure 3. Example of an t-SNE visualization and attention weight visualization of "University Hospital" infection networks. The color denotes the degree of attention weights.
Supplementary Figure 4. Example of an t-SNE visualization and attention weight visualization of "Military Units" infection networks.

Supplemental Table 1. Epidemiological survey items

Variable	Meaning	Type
id	identification number	integer
sex	gender	varchar
birthyear	year of birth	integer
nationality	nationality	varchar
residence	city of residence	varchar
confirm_date	confirmation date	date
test_date	last inspection date	date
symptom_date	date of symptom onset	date
symptom_ys	symptoms occurred (1) or not(0)	integer
cough	cough	integer
sputum	sputum	integer
sore_throat	sore throat	integer
dyspnea	shortness of breath	integer
rhinorrhea	snot	integer
fever	fever	integer
chill	chills	integer
myalgia	Muscle pain	integer
headache	headache	integer
stomache	colic	integer
diarrhea	diarrhea	integer
loss/change_taste	loss or decrease in taste	integer
loss/change_smell	loss or decrease in smell	integer
chest pain	chest pain	integer
symptom_other	other symptoms	integer
quarantine_date	quarantine start date	date
death	death(1) or not(0)	integer
death_date	date of death	date
transmission_routes	Infection route type	varchar
tr_visited_country	countries of foreign immigration	varchar
tr_cluster_type	types of clustered infection	varchar
tr_unknown	infection route is unknown	varchar

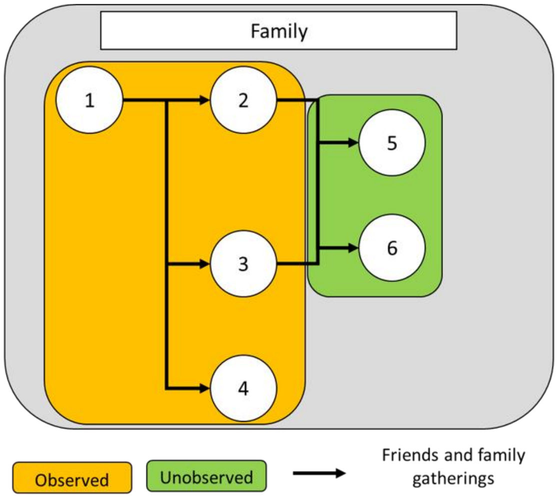

Index	Gender	Symp tom	Fever	Respi ratory	y	ŷ
1	Male	O	X	O	1	-
2	Male	O	X	O	1	1
3	Female	O	O	O	1	1
4	Male	X	X	X	0	0

Supplementary Figure 1. Example of an infection network about family gatherings. Given the observed nodes, our model, GAT-based infection prediction, predicts the infection accurately. The yellow square denotes the observed nodes, while the green denotes the unobserved variable.

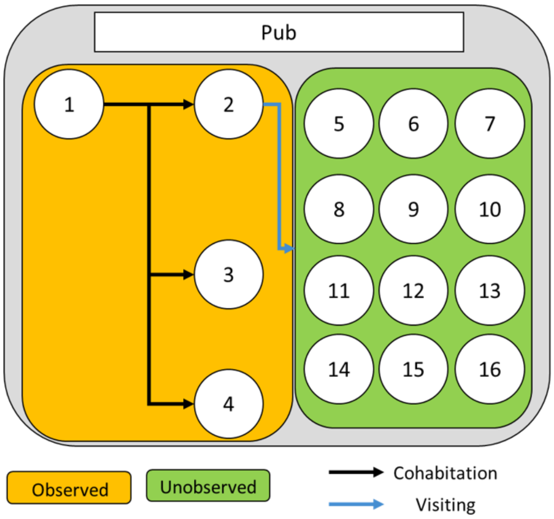

Index	Gender	Sym ptom	Fever	Respi ratory	y	ŷ
1	Female	O	O	O	-	-
2	Male	O	X	X	1	1
3	Male	O	X	O	0	1
4	Female	X	X	X	0	0

Supplementary Figure 2. Example of an infection network about pub. Given the observed nodes, our model, GAT-based infection prediction, predicts the infection accurately for nodes 2 and 4. However, our model predicts that node 3 causes additional infections, while the node 3 patient does not influence another infection.

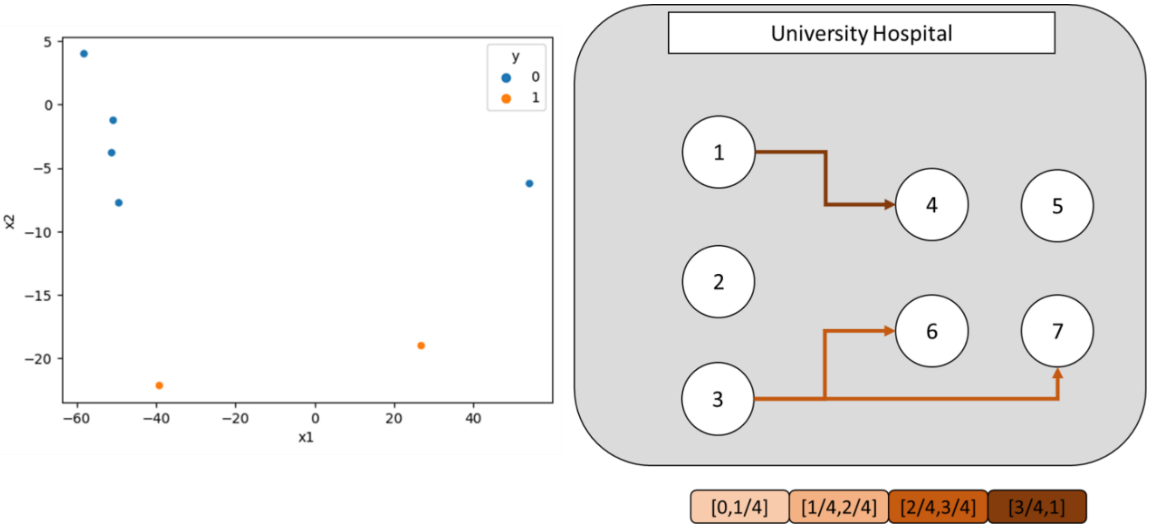

Supplementary Figure 3. Example of an t-SNE visualization and attention weight visualization of "University Hospital" infection networks. The color denotes the degree of attention weights.

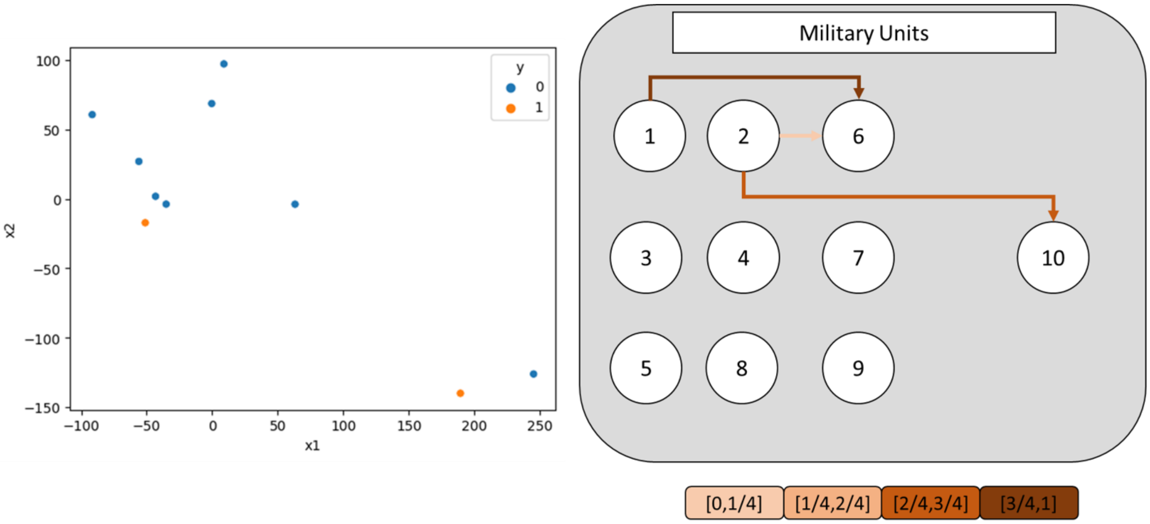

Supplementary Figure 4. Example of an t-SNE visualization and attention weight visualization of "Military Units" infection networks.
